# In vitro susceptibility of *Neisseria gonorrhoeae* to netilmicin and etimicin in comparison to gentamicin and other aminoglycosides

**DOI:** 10.1007/s10096-024-04782-2

**Published:** 2024-02-22

**Authors:** Sonja Gross, Sebastian Herren, Marina Gysin, Anna Rominski, Anna Roditscheff, Martin Risch, Frank Imkamp, David Crich, Sven N. Hobbie

**Affiliations:** 1 Institute of Medical Microbiology, University of Zurich, Gloriastrasse 30, 8006 Zurich, Switzerland; 2 Private University in the Principality of Liechtenstein, Dorfstrasse 24, 9495 Triesen, Liechtenstein; 3 Dr. Risch Medical Laboratory, Waldeggstrasse 37, 3097 Liebefeld, Switzerland; 4 Department of Pharmaceutical and Biomedical Sciences, University of Georgia, 250 West Green Street, Athens, GA 30602 USA; 5 Division of Clinical Bacteriology and Mycology, University Hospital Basel, Petersgraben 4, 4031 Basel, Switzerland

**Keywords:** Gonorrhoea, Gentamicin, Etimicin, Netilmicin, Exposure, Aminoglycoside antibiotics

## Abstract

**Purpose:**

Single doses of gentamicin have demonstrated clinical efficacy in the treatment of urogenital gonorrhea, but lower cure rates for oropharyngeal and anorectal gonorrhea. Formulations selectively enriched in specific gentamicin C congeners have been proposed as a less toxic alternative to gentamicin, potentially permitting higher dosing to result in increased plasma exposures at the extragenital sites of infection. The purpose of the present study was to compare the antibacterial activity of individual gentamicin C congeners against *Neisseria gonorrhoeae* to that of other aminoglycoside antibiotics.

**Methods:**

Antimicrobial susceptibility of three *N. gonorrhoeae* reference strains and 152 clinical isolates was assessed using standard disk diffusion, agar dilution, and epsilometer tests.

**Results:**

Gentamicin C1, C2, C1a, and C2a demonstrated similar activity against *N. gonorrhoeae*. Interestingly, susceptibility to the 1-*N*-ethylated aminoglycosides etimicin and netilmicin was significantly higher than the susceptibility to their parent compounds gentamicin C1a and sisomicin, and to any other of the 25 aminoglycosides assessed in this study. Propylamycin, a 4’-propylated paromomycin analogue, was significantly more active against *N. gonorrhoeae* than its parent compound, too.

**Conclusion:**

Selectively enriched gentamicin formulations hold promise for a less toxic but equally efficacious alternative to gentamicin. Our study warrants additional consideration of the clinically established netilmicin and etimicin for treatment of genital and perhaps extragenital gonorrhea. Additional studies are required to elucidate the mechanism behind the advantage of alkylated aminoglycosides.

**Supplementary Information:**

The online version contains supplementary material available at 10.1007/s10096-024-04782-2.

## Introduction

The Centers for Disease Control and Prevention (CDC) recommend a single dose of gentamicin as a cephalosporin-sparing alternative for the treatment of urogenital gonorrhea [1]. A number of clinical studies have consistently suggested safety and clinical efficacy of 240 to 400 mg of gentamicin intramuscular injection with cure rates ranging from 84 to 100% for urogenital gonorrhea depending on study design [2–5], whereas conflicting evidence suggested cure rates of 20 to 100% for extragenital, i.e., oropharyngeal and anorectal, gonorrhea treated with 240 to 360 mg of gentamicin alone, i.e., not in combination with azithromycin [3]. It is conceivable that higher doses and thus higher exposures of gentamicin at the relevant sites of infection may hold potential to more reliably enhance the efficacy of gentamicin including extragenital gonorrhea. However, the poor safety profile of gentamicin demands particular caution in any dose escalation strategy.

**Fig. 1:**
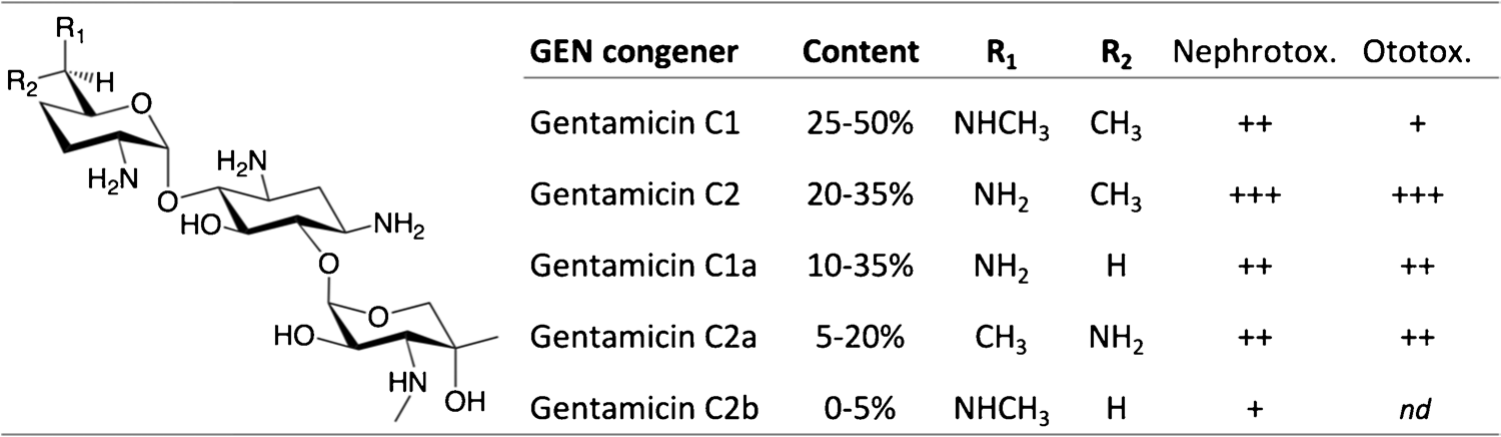
Chemical composition of gentamicin drug products and the previously reported relative toxicity of its individual congeners. The content of individual gentamicin C congeners in gentamicin drug products is defined by the United States Pharmacopeia reference standards as 25–50% gentamicin C1, 10–35% gentamicin C1a, and 25–55% of gentamicin C2 and C2a combined [31]. The contents of individual gentamicin congeners including C2, C2a, and C2b in a number and variety of gentamicin drug products have been determined previously [9, 32]. The relative nephro- and ototoxicity of each congener is ranked according to its order of magnitude reported previously, where gentamicin C2 has been the most toxic (+ + +) and C2b the least ( +) [7–9]

Gentamicin, a secondary metabolite manufactured by *Micromonospora* fermentation, is not a single chemical compound but instead is comprised of various closely related gentamicin C congeners (Fig. 1) [6]. Several studies including two recent reports have revealed or confirmed distinct toxicological profiles of individual gentamicin C congeners, suggesting that variations in composition observed for commercially available medicinal products may affect their toxicological profiles [7–9]. Some of these studies have also demonstrated relatively little difference between the four predominant congeners with regards to susceptibility of selected Gram-negative bacilli, implying that a gentamicin preparation selectively enriched in less toxic but equally potent congeners may provide additional clinical utility when compared to clinically established gentamicin products [8, 9]. However, it remains to be determined whether a selectively enriched gentamicin retains activity not only against selected bacterial strains, but also against broader panels of clinical bacterial isolates and other pathogenic species that are routinely treated with gentamicin.

Here, we used disk diffusion, agar dilution, and epsilometer (*E *test) antimicrobial susceptibility testing to study the antibacterial activity of individual gentamicin C congeners against *Neisseria gonorrhoeae* in comparison to gentamicin and other aminoglycoside antibiotics. Compounds of interest were assessed with a larger panel of clinical *N. gonorrhoeae* isolates including multidrug-resistant isolates, to identify a safer and more active alternative to gentamicin for dose escalation studies in the treatment of extragenital gonorrhea.

## Materials and methods

### Bacterial strains and culture conditions

Bacterial strains and clinical isolates used in this study are summarized in Tables S1 and Table S2, respectively. Some of the isolates have been characterized before, and some were known to be resistant to ceftriaxone (CTR), azithromycin (AZI), or any combination of several other drugs [10]. Bacterial culture conditions were selected according to the Clinical and Laboratory Standards Institute (CLSI) reference methodology M07 [11].

### Antimicrobial susceptibility testing

Kirby–Bauer disk diffusion and agar dilution assays largely followed the CDC Gonorrhea Laboratory Information guideline (https://www.cdc.gov/std/gonorrhea/lab/testing.htm), which is based on CLSI reference methodologies M07 and performance standards M100 [11, 12], with small adjustments. In brief, approximately 10^4^ colony forming units (CFU) were spotted on chocolate agar containing a defined concentration of antibiotic. The MIC was defined as the lowest concentration that fully inhibited growth during 20 to 24 h of incubation at 37 °C. The quality control strain ATCC 49226 (strain F-15) was tested in every experiment and results compared against the performance standard. The *Neisseria meningitidis* strain ATCC 13077 was included as an experimental outgroup reference. For disk diffusion, a cell suspension at 0.5 McFarland standard was evenly spread with a sterile cotton swab onto chocolate agar plates. Blank disks (Sensi-Disc, BD) were applied to inoculated agar plates and customized by adding the relevant amounts of antibiotic to a disk. Where available, commercial antibiotic disks (Oxoid, Thermo; Liofilchem) were applied to the inoculated agar plates for comparison. Inhibition zone diameters were determined after 20 to 24 h incubation at 37 °C. The quality control strain ATCC 49226 was tested in every experiment with a 30-μg ceftriaxone disk for comparison against the performance standard. *E* tests were done according to manufacturers’ instructions. Although *E* tests are not a validated method for diagnostic susceptibility testing, we opted to selectively use *E* tests as an additional source of experimental susceptibility data. In accordance with the referenced guidelines, the incubator atmosphere routinely contained 5% CO_2_ across all regular susceptibility testing. Incubation without CO_2_ would result in a less acidic medium and thus affect the susceptibility readouts, as we confirmed in a preliminary experiment (Fig. S1).

### Subcontracted susceptibility testing with CDC isolates

Antimicrobial susceptibility testing of 96 CDC isolates (Table S2) by the agar dilution method was subcontracted to Southern Research in Birmingham, AL, USA. Rectangular agar plates (Thermo Scientific, Nunc 267,060) were prepared containing 3.6% GC agar base (Oxoid, OXCM0367B), 1% defined growth supplement (BD, BBL IsoVitaleX, B11876), and a twofold serial dilution of antibiotic consisting of 12 compound dilution steps. The serial dilutions in agar medium were prepared in triplicate. The concentration ranges were 16–0.008 μg/mL for propylamycin, azithromycin, penicillin, and tetracycline; 256–0.125 μg/ml for gentamicin and apramycin; 4–0.002 μg/mL for ceftriaxone and cefixime; and 32–0.016 μg/mL for ciprofloxacin. Strains were grown on drug-free agar plates (BD, BBLTM Chocolate II Agar plates) for 24 h at 36 ± 1 °C in 5% CO_2_. Colonies were suspended directly in 0.9% saline supplemented with 0.5 × Mueller Hinton medium. In preparation for the MIC assay, the inocula were standardized by adjusting their turbidity to the 0.5 McFarland standard and transferred by dispensing 700-μl aliquots to 96-well deep well plates. The deep well plates with the inocula were maintained on ice. Replicators with 96 × 1.5 mm pins (Boekel Industries, #140500) were sterilized in an autoclave before the start of the experiment and with an open flame between the transfer steps. The use of multiple replicators ensured that the transfer pins had cooled down to ambient temperature before every transfer performed during the experiment. The 96-pin replicators were used to transfer 1-μl aliquots of bacterial suspension from the inocula-containing 96-well deep well plates to the agar surface of the assay plates containing test article or control antibiotic. The same method was used to inoculate compound-free agar plates for growth control. Plates were incubated at 36 ± 1 °C in 5% CO_2_, for 24 h as per diagnostic protocol and up to a total of 48 h experimentally. After the incubation period, assay and growth control plates were inspected visually, and the MIC was recorded as the lowest concentration of compound that completely inhibited growth.

### Frequency of resistance studies

To determine the rate of spontaneous resistance mutation, approximately 10^8^ CFU were plated onto selective chocolate agar plates containing antibiotic concentrations at 2, 4, or 6 times the MIC. A serial tenfold dilution of the inoculum was also plated on non-selective agar plates to determine the total number of CFUs in the inoculum. The inocula were evenly spread and incubated for 48 h at 37 °C, as there was no visible growth on any of the selective agar plates after 24 h. CFU on the selective plates were counted and divided by the effective number of CFUs in the inoculum to determine the frequency of resistance.

### Statistical analysis

Assuming a non-Gaussian distribution of antibiotic susceptibilities to account for the possibility of drug resistant isolates, the Mann–Whitney *U* test, a nonparametric Wilcoxon rank-sum test (WSR), was applied for statistical analysis.

## Results

**Fig. 2:**
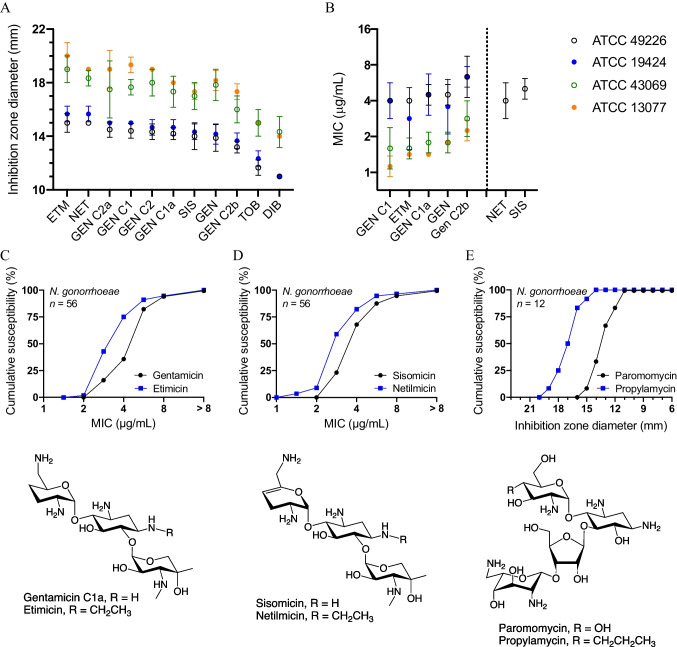
In vitro activities of aminoglycoside antibiotics against *Neisseria* spp. **A** Kirby–Bauer susceptibility testing results (mean ± SD, *n* ≥ 3) using 10-μg content disks and the three *N. gonorrhoeae* reference strains ATCC 49226 (strain F-18), 43,069 (CDC Ng-116), and 19,424 (NCTC 8375); as well as the *N. meningitidis* reference strain ATCC 13077 for experimental comparison. **B** Agar dilution susceptibility testing results for the same four reference strains (geometric mean ± SD, *n* = 3). **C**, **D** Cumulative proportion of isolates with an MIC equal to or smaller than the MICs indicated on the abscissa, for 56 clinical *N. gonorrhoeae* isolates based on agar dilution susceptibility testing comparing etimicin to gentamicin (C) and netilmicin to sisomicin (D), respectively. **E** Cumulative susceptibility of twelve clinical isolates by propylamycin and paromomycin. The chemical structures are depicted to highlight the alkylations in etimicin, netilmicin, and propylamycin. ETM, etimicin; NET, netilmicin; GEN, gentamicin; SIS, sisomicin; GEN C2b, micronomicin; TOB, tobramycin; DIB, dibekacin

We first assessed the susceptibility of the three *N. gonorrhoeae* reference strains F-15 (ATCC 49226), CDC Ng-116 (ATCC 43069) and NCTC 8375 (ATCC 19424) using the Kirby–Bauer disk diffusion assay (Fig. 2A). Strain CDC Ng-116 appeared to be generally more susceptible to aminoglycoside antibiotics than the other two strains. The antibacterial potency of individual gentamicin congeners was similar to that of the clinical gentamicin complex. Interestingly, etimicin and netilmicin appeared to be the most active aminoglycosides, more active than their parent compounds gentamicin C1a and sisomicin, respectively, and more active than the various other gentamicin C congeners. Tobramycin and dibekacin were less potent than gentamicin. For a comprehensive assessment of *N. gonorrhoeae* susceptibility to aminoglycosides, we tested a wider panel of aminoglycosides that included amikacin, plazomicin, arbekacin, isepamicin, apramycin, neomycin B, ribostamycin, paromomycin, kanamycin, and kanamycin B, all of which showed lower activity than the gentamicins as well (Fig. S2).

Next, we used the agar dilution method to corroborate the disk diffusion results for a few selected aminoglycosides (Fig. 2B). The MICs again indicated a similar potency of the individual gentamicin congeners with etimicin appearing slightly more potent than gentamicin C1a, gentamicin C1 being one of the more active constituents, and micronomicin, a fraction enriched in gentamicin C2b, one of the less active. The difference in activity between gentamicin C1a and its 1-*N*-ethylated analogue etimicin prompted us to subsequently also determine the agar dilution MICs of sisomicin and its 1-*N*-ethylated analogue netilmicin for the quality control reference strain F-15, likewise revealing a higher activity of the alkylated derivative when compared to the parent compound.

Since we considered the relative potencies of the clinically established netilmicin and etimicin as an encouraging observation, we decided to study a panel of 56 drug-resistant *N. gonorrhoeae* clinical isolates in an agar dilution susceptibility test (Fig. 2C–D). Of the 56 clinical isolates, 42 and 46 (≥ 75%) displayed etimicin and netilmicin MICs of ≤ 4 mg/mL, respectively, as opposed to only 20 (36%) for gentamicin and 38 (68%) for sisomicin. The geometric mean of the etimicin MIC was 3.56 μg/mL and thus significantly lower than the mean gentamicin MIC of 5.26 μg/mL (*p* < 0.0001, Table S3). The mean netilmicin MIC was 2.85 μg/mL in comparison to a mean sisomicin MIC of 4 μg/mL (*p* < 0.001).

**Fig. 3:**
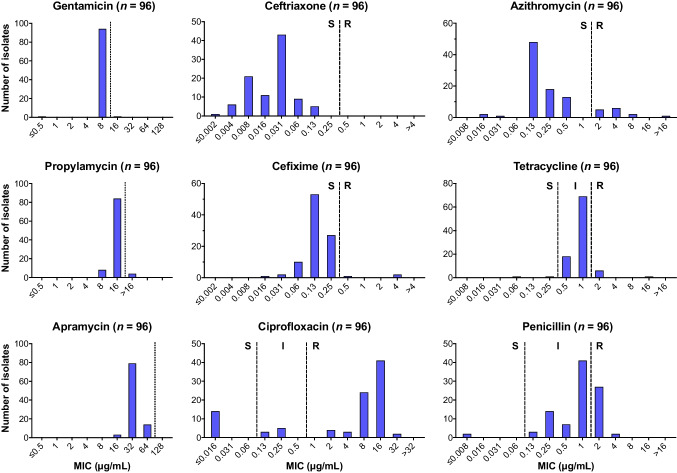
MIC distributions of propylamycin and apramycin in comparison to gentamicin and other gonorrhea antibiotics. Clinical breakpoints for gentamicin have not been defined. Vertical dotted lines indicate the MIC_90_ cut-off for the three aminoglycosides. For all other drugs, vertical dashed lines indicate the CLSI interpretative criteria for the susceptible (S), intermediate (I), and resistant (R) subpopulations [12]

The difference in chemical structures between etimicin and netilmicin and their parent compounds gentamicin C1a and sisomicin is depicted in Fig. 2C–D, highlighting the 1-*N*-ethylation as the only structural difference in both pairs. To assess whether the beneficial effect of alkylation is specific to the 1-amino group in gentamicin and sisomicin, we decided to also test the antibacterial potency of propylamycin, a 4’-proplyated derivative of paromomycin [13], against *N. gonorrhoeae*. Interestingly, the intrinsically low activity of paromomycin against twelve *N. gonorrhoeae* clinical isolates was greatly enhanced by a 4’-propyl modification, from a mean inhibition zone diameter of 12.9 to 16.6 mm (*p* < 0.00001) (Fig. 2E). This remarkable difference led us to subcontract another agar dilution susceptibility test to determine the propylamycin susceptibility of 96 clinical *N. gonorrhoeae* isolates in comparison to gentamicin, apramycin, and non-aminoglycoside standard of care drugs. The MIC distributions revealed the propylamycin potency (geometric mean of MIC = 15.5 μg/mL) was about twofold lower than that of gentamicin (7.8 μg/mL), but twofold higher than that of apramycin (34.6 μg/mL) (Fig. 3).

Since gentamicin is primarily considered a second-line therapeutic option of interest in cases of drug-resistant gonorrhea, we considered the potential of resistance to gentamicin itself to be of relevance as well. For three of the collectively 152 isolates studied, the gentamicin MIC was determined to be > 8 μg/mL by agar dilution, but did not show any significantly reduced susceptibility to gentamicin with the *E* test or disk diffusion assays (Table S2). The frequency of spontaneous *N. gonorrhoeae* resistance to individual gentamicin congeners was determined as < 7.8 × 10^−9^, which was not significantly different from the frequency observed for gentamicin (Table S4).

## Discussion

Our study demonstrates that there is no significant difference between individual gentamicin C congeners with regards to their potency against *Neisseria gonorrhoeae*, with the exception of gentamicin C2b, which appears to be the least potent congener. The gentamicin congeners C1, C2, C1a, and C2a appeared slightly more potent than the natural gentamicin complex currently being used in the clinics. These findings confirm similar observations previously reported for other Gram-negative pathogens, and is in support of proposals of gentamicin formulations selectively enriched in less toxic but equally potent congeners, for instance by removal of gentamicin C2 from the natural gentamicin complex [8, 9]. Apart from chromatographic separation, chemical synthesis of individual gentamicin C congeners may provide an alternative strategy [14].

We further found that two aminoglycoside antibiotics in clinical use, netilmicin and etimicin, are more potent than gentamicin or any of its individual congeners, warranting further consideration in designing future treatment regimens for gonorrhea. Netilmicin and etimicin have not established themselves as standard-of-care drugs in clinical practice, probably because they provide little therapeutic benefit over gentamicin, tobramycin, or amikacin in the treatment of Gram-negative bacilli infections. For *N. gonorrhoeae*, however, our data suggest higher susceptibility to netilmicin and etimicin than to the current standard of care gentamicin, or any other aminoglycoside (Fig. 2, Fig. S2). Our in vitro results compare well to the reported clinical efficacy of netilmicin in the treatment of gonorrhea [15, 16]. The potency of the clinical antibiotics tobramycin (Fig. 2A) and amikacin (Fig. S2) was particularly low for *N. gonorrhoeae* in our studies.

Comparing the relative activities of more than 20 aminoglycosides provides interesting insights into the structure–activity relationship. Dibekacin (Fig. S3) has a kanosamine instead of a garosamine at position 6 of the 2-deoxystreptamine, but is otherwise identical in structure to gentamicin C1a (Fig. 2C). This difference appears to significantly reduce the molecule’s activity against *N. gonorrhoeae* (Fig. 2A). The key structural differences are the two methyl groups in garosamine that are absent in kanosamine. The lower activity of dibekacin appears to be partly offset by a 4’-oxygenation as in tobramycin, but apparently not by a 3’,4’-dioxygenation as in kanamycin B, kanamycin A, or amikacin; nor by a 1-acylation as in arbekacin (Fig. 2A, Fig. S2, Fig. S3).

The anti-gonococcal activity of sisomicin (Fig. 2D) is not significantly different from that of gentamicin C1a (Fig. 2C), suggesting the 4’,5’-didehydration in sisomicin has little effect. The 1-*N*-ethylation in netilmicin and etimicin, however, increased the potency of the parent molecules sisomicin and gentamicin C1a (Fig. 2C, D). The mechanism behind this improvement is currently unclear, but one may speculate that the additional lipophilicity (XLogP3-AA of -4.2, compared to -5.1 and -5.0, respectively) may impact cellular drug uptake or efflux in *N*. *gonorrhoeae*. In contrast, the 1-acylations in the 6-garosamine compounds plazomicin and isepamicin appear to reduce the potency, although a direct comparison is difficult because of the additional 2’-deamino-2’,3’,4’-trioxygenation in isepamicin and 6-*N*-acylation in plazomicin.

If the alkylations found in the garosamine ring and as substituents in the 2-deoxystreptamine were to positively affect the anti-gonococcal activity by means of the changes in their physicochemical properties rather than by molecular interactions in the drug target site, then we expected the positive effect of an alkyl group to also benefit aminoglycosides of distinct chemical structure, i.e., in the 4,5-disubtituted as well as the 4,6-disubstituted 2-deoxstreptamines. Indeed we found that the 4’-deoxy-4’propyl paromomycin derivative propylamycin (XLogP-AA of -7.0) [13], is significantly more active against *N. gonorrhoeae* than the parent molecule paromomycin (XLogP-AA of − 8.7) (Fig. 2E). This observation supports the hypothesis that the introduction of an alkyl group into aminoglycosides more generally benefits the anti-gonococcal activity through physicochemical parameters and warrants additional studies into the mechanisms of cell wall permeability and intracellular drug accumulation. The findings presented here may also provide a starting point for further lead optimization by medicinal chemistry.

Gentamicin has a relatively poor safety profile, limiting the potential for therapeutic dose escalation in targeting extragenital gonorrhea. Gentamicin is comprised of 20–35% of gentamicin C2, which has been identified as the most toxic of the individual congeners (Fig. 1) [8, 9]. Interestingly, its stereoisomer gentamicin C2a was found to be less toxic, but chromatographic separation of the two isomers is difficult, so one may envision a gentamicin formulation enriched in C1 and C1a, with all C2 congeners removed. Animal studies have revealed gentamicin C1 and C1a to be less toxic than the gentamicin complex [8, 9, 17], as is their 1-*N*-ethyl derivative etimicin [18]. Clinical safety data for etimicin and netilmicin have been reported [19].

Propylamycin has been designed to increase the ribosomal target selectivity for bacterial over mitochondrial ribosomes, and has indeed demonstrated an exceptional safety profile when compared to other aminoglycosides [13]. A favorable safety profile has also been demonstrated for apramycin, an unusual octadiose aminoglycoside currently in clinical development for the treatment of infections caused by Gram-negative bacilli [17, 20–22]. Kirby and co-workers have previously reported encouraging apramycin activity against 72 *N. gonorrhoeae* clinical isolates, including spectinomycin-resistant strains, with an epidemiological cut-off of 64 μg/mL [23]. Our present data on apramycin for additional multidrug resistant isolates agrees with those findings, and further underscores the unique potential of apramycin (Fig. 3). Although propylamycin and apramycin are twofold and fourfold less active than gentamicin, respectively, their lower toxicity may allow for high enough dosing to attain efficacious exposures not only in the urogenital tract, but also at the extragenital sites of infection. It may also be possible to further improve the anti-gonococcal activity of apramycin by alkylation. However, additional studies are required to further explore and assess the potential for additional optimization by medicinal chemistry.

In addition to the reported efficacy and safety of single-dose intramuscular gentamicin, another rationale in favor of this drug has been a virtual lack of clinical gentamicin resistance to date. To our best knowledge, true clinical gentamicin resistance by horizontal gene transfer has not been reported yet for *N. gonorrhoeae*. Rare cases of reduced gentamicin susceptibility have been characterized by only small increases in MIC to ≤ 32 μg/mL, i.e., a phenotype similar to two of our isolates [24, 25]. We have searched the literature for truly gentamicin-resistant clinical *N. gonorrhoeae* isolates with little success. Very few isolates originally reported as resistant with gentamicin MICs of > 32 μg/mL were communicated to not be reproducible in repeat susceptibility testing when using *E* tests instead of agar dilution [26–28], an observation supported by our own findings as well. In vitro selection pressure and passaging experiments have recently identified mutations in the *fusA* gene, coding for the ribosomal elongation factor G, as possible gentamicin resistance determinants in *N. gonorrhoeae*, but the clinical relevance of such laboratory-induced mutations has yet to be elucidated [29, 30].

## Conclusion

Netilmicin, etimicin, or gentamicin preparations selectively enriched in gentamicin C1 and C1a may provide equally effective but safer therapeutics than gentamicin for the treatment of gonorrhea with a cephalosporin-sparing regimen. In vivo studies are warranted to support PKPD target attainment modeling in support of a higher aminoglycoside dose in the treatment of oropharyngeal and anorectal gonorrhea. The beneficial effect of aminoglycoside alkylation may provide an important clue for further lead optimization programs specifically targeting gonorrhea.

### Supplementary Information

Below is the link to the electronic supplementary material.

Supplementary file1 (PDF 577 KB)

## Data Availability

All data supporting the findings of this study are available within the article and its supplementary data files.
