# Supplementary material for: In vitro susceptibility of Neisseria gonorrhoeae to netilmicin and etimicin in comparison to gentamicin and other aminoglycosides
Source: Eur J Clin Microbiol Infect Dis. 2024 Feb 22;43(5):821–8. doi: 10.1007/s10096-024-04782-2 (PMC11108870; doi:10.1007/s10096-024-04782-2)
Supplement: Supplementary file 1 — Supplementary file1 (PDF 577 KB) [file 10096_2024_4782_MOESM1_ESM.pdf]

In-vitro susceptibility of *Neisseria gonorrhoeae* to netilmicin and etimicin in comparison to gentamicin and other aminoglycosides

Sonja Gross, Sebastian Herren, Marina Gysin, Anna Rominski, Anna Roditscheff, Martin Risch, Frank Imkamp, David Crich, Sven N. Hobbie

Supplementary Information

Table S1 List of bacterial reference strains used in this study

		Agar Dilution, MIC (µg/mL), geometric mean												
**Species**	**Strain**	**GEN**	**C1**	**C1a**	**C2b**	**ETM**	**SIS**	**NET**	**APR**	**CTR**	**AZI**	**CTZ**	**SOL**	**ZOL**
N. gonorrhoeae	ATCC 49226	5.1	4.0	4.5	6.3	4.5	5.0	4.0	16	0.016	0.42	0.125	0.25	0.25
N. gonorrhoeae	ATCC 19424	3.6	4.0	4.5	6.3	2.8	*nd*	*nd*	*nd*	*nd*	*nd*	*nd*	*nd*	*nd*
N. gonorrhoeae	ATCC 43069	1.8	1.6	1.8	2.8	1.6	*nd*	*nd*	*nd*	*nd*	*nd*	*nd*	*nd*	*nd*
N. meningitidis	ATCC 13077	1.8	1.1	1.4	2.2	1.4	*nd*	*nd*	*nd*	*nd*	*nd*	*nd*	*nd*	*nd*
CLSI QC range *N. gonorrhoeae*		4-16	-	-	-	-	-	-	-	0.004-0.016	0.25-1	-	0.03-0.25	0.06-0.5
CLSI Breakpoint *N. gonorrhoeae*		-	-	-	-	-	-	-	-	≤ 0.025	≤ 1	-	-	-

		Disk diffusion, inhibition zone diameter (mm,) arithmetic mean													
		**GEN30**	**GEN10**	**C1 10**	**C1a 10**	**C2 10**	**C2a 10**	**C2b 10**	**ETM10**	**SIS10**	**NET10**	**TOB10**	**DIB10**	**AMK10**	**APR10**
N. gonorrhoeae	ATCC 49226	18.0	13.9	14.7	14.7	14.8	14.5	13.5	15.2	14.3	15.0	12.8	10.8	7.0	8.0
N. gonorrhoeae	ATCC 19424	18.3	14.2	15.0	14.7	14.7	15.0	13.7	15.7	14.3	15.7	12.3	11.0	7.0	9.0
N. gonorrhoeae	ATCC 43069	22.0	17.8	17.7	17.3	18.0	17.5	16.0	19.0	17.0	18.3	15.0	14.3	11.0	12.0
N. meningitidis	ATCC 13077	21.7	18.2	19.3	18.0	19.0	19.0	17.3	20.0	17.3	19.0	15.0	14.0	14.0	13.0

		Etest, MIC (µg/mL), geometric mean								
		**GEN**	**CTR**	**AZI**	**CIX**	**CPO**	**CIP**	**ERT**	**PEN**	**TET**
N. gonorrhoeae	ATCC 49226	3.4	≤0.016	0.19	≤0.016	0.094	0.002	0.006	0.5	1.1
N. gonorrhoeae	ATCC 19424	3.5	≤0.016	*nd*	*nd*	≤0.016	*nd*	*nd*	*nd*	*nd*
N. gonorrhoeae	ATCC 43069	2.5	≤0.016	*nd*	*nd*	≤0.016	*nd*	*nd*	*nd*	*nd*
N. meningitidis	ATCC 13077	2.1	≤0.016	*nd*	*nd*	≤0.016	*nd*	*nd*	*nd*	*nd*

AMK, amikacin; APR, apramycin; AZI, azithromycin; C1, gentamicin C1; C1a, gentamicin C1a; C2, gentamicin C2; C2a, gentamicin C2a; C2b, micronomicin; CIP, ciprofloxacin; CIX, cefixime; CPO, cefpodoxime; CTR, ceftriaxone; CTZ, ceftazidime; DIB, dibekacin; ERT, ertapenem; ETM, etimicin; GEN, gentamicin; NET, netilmicin; PEN, penicillin; SIS, sisomicin; SOL, solithromycin; TET, tetracycline; TOB, tobramycin; ZOL, zoliflodacin.

Table S2 List of bacterial clinical isolates used in this study

		MIC (µg/mL)							Inhibition zone (mm)	
		Agar Dilution (geometric mean)						Etest	Disk diffusion	
**Species**	**Isolate**	**GEN**	**ETM**	**SIS**	**NET**	**CTR**	**AZI**	**GEN**	**GEN30**	**TOB10**
N. gonorrhoeae	IMM 2019100109	>8	>8	>8	>8	0.003	0.5	6	*nd*	*nd*
N. gonorrhoeae	IMM 2019102452	2.8	2.8	5.7	2.8	0.008	1.4	4	*nd*	*nd*
N. gonorrhoeae	IMM 2019102504	2.8	2.8	5.7	2.8	0.016	0.25	3	*nd*	*nd*
N. gonorrhoeae	IMM 2019102600	4.0	2.8	4.0	2.8	0.016	>16	4	*nd*	*nd*
N. gonorrhoeae	IMM 2019102604	5.7	2.8	4.0	2.8	0.008	0.25	4	*nd*	*nd*
N. gonorrhoeae	IMM 2019102621	2.8	2.0	2.8	2.0	0.011	0.125	2	*nd*	*nd*
N. gonorrhoeae	IMM 2020100033	2.8	2.8	2.8	2.0	0.5	0.5	3	*nd*	*nd*
N. gonorrhoeae	IMM 2020100103	>8	>8	>8	>8	0.008	0.125	8	*nd*	*nd*
N. gonorrhoeae	IMM 2020830221	2.8	2.8	2.8	2.0	0.008	0.063	3	*nd*	*nd*
N. gonorrhoeae	IMM 2020100137	2.8	2.8	2.8	1.4	0.008	0.063	3	*nd*	*nd*
N. gonorrhoeae	IMM 2020100222	5.7	5.7	5.7	5.7	0.016	0.35	6	*nd*	*nd*
N. gonorrhoeae	IMM 2020100257	2.8	2.8	2.8	2.8	0.008	≤0.03	2	*nd*	*nd*
N. gonorrhoeae	IMM 2020100415	5.7	2.8	2.8	2.8	0.008	2.0	4	*nd*	*nd*
N. gonorrhoeae	IMM 2020100548	4.0	2.8	4.0	2.8	0.004	0.5	3	*nd*	*nd*
N. gonorrhoeae	IMM 2020100570	4.0	2.8	4.0	2.8	0.016	0.25	3	*nd*	*nd*
N. gonorrhoeae	IMM 2020100695	2.8	2.8	4.0	2.8	0.004	2.0	4	*nd*	*nd*
N. gonorrhoeae	IMM 2020100916	4.0	2.8	2.8	2.8	0.022	0.25	3	*nd*	*nd*
N. gonorrhoeae	IMM 2020100953	5.7	4.0	4.0	2.8	0.031	0.5	3	*nd*	*nd*
N. gonorrhoeae	IMM 2020101087	5.7	4.0	4.0	4.0	0.008	2.0	4	*nd*	*nd*
N. gonorrhoeae	IMM 2020101116	5.7	4.0	4.0	4.0	0.011	2.0	4	*nd*	*nd*
N. gonorrhoeae	IMM 2020101188	5.7	2.8	2.8	2.8	0.004	0.125	3	*nd*	*nd*
N. gonorrhoeae	IMM 2020101615	4.0	2.8	2.8	2.8	0.011	>16	4	*nd*	*nd*
N. gonorrhoeae	IMM 2020102022	5.7	4.0	4.0	2.8	0.008	2.0	4	*nd*	*nd*
N. gonorrhoeae	IMM 2020102140	4.0	2.8	4.0	2.8	0.008	2.0	4	*nd*	*nd*
N. gonorrhoeae	IMM 2020102312	5.7	4.0	2.8	2.8	0.016	0.25	3	*nd*	*nd*
N. gonorrhoeae	IMM 2020102573	5.7	4.0	2.8	2.8	0.011	0.25	3	*nd*	*nd*
N. gonorrhoeae	IMM 2020102701	5.7	2.8	2.8	2.8	0.011	0.25	2	*nd*	*nd*
N. gonorrhoeae	IMM 2021100328	8.0	5.7	5.7	5.7	0.016	4.0	6	*nd*	*nd*
N. gonorrhoeae	IMM 2021100727	5.7	2.8	4.0	2.8	0.016	0.25	3	*nd*	*nd*
N. gonorrhoeae	IMM 2021101006	2.8	2.8	4.0	1.4	0.008	0.125	3	*nd*	*nd*
N. gonorrhoeae	IMM 2021101089	4.0	2.8	4.0	2.8	0.004	0.25	3	*nd*	*nd*
N. gonorrhoeae	IMM 2021101178	4.0	2.8	4.0	2.8	0.016	0.25	3	*nd*	*nd*
N. gonorrhoeae	IMM 2021101301	4.0	2.8	4.0	2.8	0.003	1.0	3	*nd*	*nd*
N. gonorrhoeae	IMM 2021102029	4.0	2.8	2.8	2.8	0.031	1.0	3	*nd*	*nd*
N. gonorrhoeae	IMM 2015101152	4.0	4.0	4.0	2.8	*nd*	*nd*	*nd*	21	13
N. gonorrhoeae	IMM 2015101176	5.7	4.0	4.0	4.0	*nd*	*nd*	*nd*	28	22
N. gonorrhoeae	IMM 2015101189	8.0	8.0	8.0	5.7	*nd*	*nd*	*nd*	20	13
N. gonorrhoeae	IMM 2015101369	5.7	4.0	5.7	4.0	*nd*	*nd*	*nd*	19	12
N. gonorrhoeae	IMM 2016100004	8.0	8.0	8.0	5.7	*nd*	*nd*	*nd*	17	12
N. gonorrhoeae	IMM 2016100103	5.7	4.0	4.0	4.0	*nd*	*nd*	*nd*	21	14
N. gonorrhoeae	IMM 2016100125	8.0	5.7	5.7	4.0	*nd*	*nd*	*nd*	19	14
N. gonorrhoeae	IMM 2016100126	5.7	4.0	5.7	4.0	*nd*	*nd*	*nd*	19	13
N. gonorrhoeae	IMM 2016100134	8.0	5.7	8.0	5.7	*nd*	*nd*	*nd*	21	13
N. gonorrhoeae	IMM 2016100488	5.7	4.0	4.0	4.0	*nd*	*nd*	*nd*	19	11
N. gonorrhoeae	IMM 2016100846	5.7	5.7	5.7	4.0	*nd*	*nd*	*nd*	17	13
N. gonorrhoeae	IMM 2016100994	>8	>8	>8	8.0	*nd*	*nd*	*nd*	19	12
N. gonorrhoeae	RL GN_24	5.7	4.0	4.0	4.0	*nd*	*nd*	*nd*	*nd*	*nd*
N. gonorrhoeae	RL GN_61	5.7	4.0	4.0	4.0	*nd*	*nd*	*nd*	*nd*	*nd*
N. gonorrhoeae	RL GN_83	8.0	5.7	8.0	5.7	*nd*	*nd*	*nd*	*nd*	*nd*
N. gonorrhoeae	RL GN_95	5.7	4.0	5.7	2.8	*nd*	*nd*	*nd*	*nd*	*nd*
N. gonorrhoeae	RL GN_110	5.7	4.0	4.0	4.0	*nd*	*nd*	*nd*	*nd*	*nd*
N. gonorrhoeae	RL GN_aa181	5.7	4.0	4.0	2.8	*nd*	*nd*	*nd*	*nd*	*nd*
N. gonorrhoeae	RL GN_182	8.0	5.7	5.7	5.7	*nd*	*nd*	*nd*	*nd*	*nd*
N. gonorrhoeae	RL GN_aa183	5.7	5.7	4.0	2.8	*nd*	*nd*	*nd*	*nd*	*nd*
N. gonorrhoeae	RL GN_585	5.7	5.7	5.7	4.0	*nd*	*nd*	*nd*	*nd*	*nd*
N. gonorrhoeae	RL GN_588	5.7	4.0	4.0	2.8	*nd*	*nd*	*nd*	*nd*	*nd*

AZI, azithromycin (S ≤1); CTR, ceftriaxone (S ≤0.025); ETM, etimicin; GEN, gentamicin (S ≤16); GEN30, 30 µg of gentamicin; NET, netilmicin; SIS, sisomicin; TOB10, 10 µg of tobramycin.

Table S2 (continued)

Species	Strain	MIC (µg/mL), Agar Dilution								
		GEN	PRP	APR	CTR	AZI	CIX	PEN	TET	CIP
N. gonorrhoeae	CDC01	8	16	32	0.031	0.25	0.125	1	2	8
N. gonorrhoeae	CDC02	8	16	32	0.031	0.5	0.125	1	1	16
N. gonorrhoeae	CDC03	8	16	32	0.031	0.125	0.125	1	1	8
N. gonorrhoeae	CDC04	8	16	32	0.031	0.125	0.125	1	0.5	8
N. gonorrhoeae	CDC05	8	16	32	0.063	0.5	0.125	1	1	8
N. gonorrhoeae	CDC06	8	16	32	0.063	0.5	0.25	1	1	8
N. gonorrhoeae	CDC08	8	16	32	0.016	0.125	0.125	0.25	1	8
N. gonorrhoeae	CDC09	8	16	32	0.031	0.125	0.125	1	1	8
N. gonorrhoeae	CDC10	8	16	64	0.031	0.5	0.125	1	1	16
N. gonorrhoeae	CDC11	8	16	32	0.031	0.25	0.125	1	1	8
N. gonorrhoeae	CDC12	8	16	32	0.031	0.125	0.25	1	1	8
N. gonorrhoeae	CDC13	8	16	32	0.031	0.125	0.063	1	1	8
N. gonorrhoeae	CDC14	8	16	32	0.031	0.125	0.25	1	1	8
N. gonorrhoeae	CDC15	8	16	32	0.008	4	0.031	0.125	0.5	2
N. gonorrhoeae	CDC16	8	16	32	0.063	0.25	4	2	1	8
N. gonorrhoeae	CDC18	8	>16	64	0.063	0.5	0.063	1	1	16
N. gonorrhoeae	CDC19	8	16	32	0.008	8	0.125	0.25	0.5	<0.016
N. gonorrhoeae	CDC20	8	8	16	0.063	>16.0	0.125	0.125	1	2
N. gonorrhoeae	CDC21	8	16	32	0.031	0.125	0.25	1	1	16
N. gonorrhoeae	CDC22	8	16	32	0.008	0.5	0.031	0.25	1	4
N. gonorrhoeae	CDC23	8	>16	64	0.031	0.125	0.125	1	1	8
N. gonorrhoeae	CDC24	8	16	32	0.063	0.25	0.063	1	1	8
N. gonorrhoeae	CDC25	8	16	32	0.031	0.125	0.25	2	1	8
N. gonorrhoeae	CDC26	8	16	32	0.031	0.5	0.063	0.25	0.5	4
N. gonorrhoeae	CDC27	8	16	32	0.031	0.25	0.063	1	1	8
N. gonorrhoeae	CDC28	8	16	32	0.031	0.25	0.25	1	1	16
N. gonorrhoeae	CDC29	8	16	32	0.016	0.5	0.125	1	1	<0.016
N. gonorrhoeae	CDC30	8	16	32	0.031	0.25	0.125	1	1	8
N. gonorrhoeae	CDC31	8	16	32	0.031	0.5	0.25	2	2	8
N. gonorrhoeae	CDC32	8	16	16	0.063	0.125	0.125	1	0.5	8
N. gonorrhoeae	CDC33	8	8	16	0.125	0.125	0.5	1	0.5	2
N. gonorrhoeae	CDC34	8	16	32	0.125	0.125	0.063	1	1	16
N. gonorrhoeae	CDC35	8	16	32	0.125	0.25	0.25	2	2	16
N. gonorrhoeae	CDC36	8	8	32	0.031	4	0.063	0.25	1	8
N. gonorrhoeae	CDC37	8	>16	64	0.031	0.25	0.125	1	1	16
N. gonorrhoeae	CDC38	8	>16	32	0.031	0.25	0.063	1	1	16
N. gonorrhoeae	CDC39	8	16	32	0.031	0.25	0.25	2	1	8
N. gonorrhoeae	CDC40	8	16	32	0.031	0.25	0.125	1	1	8
N. gonorrhoeae	CDC41	8	16	64	0.031	0.25	0.25	2	1	8
N. gonorrhoeae	CDC42	8	16	32	0.031	0.25	0.25	2	2	16
N. gonorrhoeae	CDC43	8	16	32	0.031	0.25	0.25	2	2	16
N. gonorrhoeae	CDC45	8	16	32	0.125	0.5	0.25	2	2	16
N. gonorrhoeae	CDC46	8	16	32	0.031	0.125	0.125	1	1	16
N. gonorrhoeae	CDC47	8	16	32	0.031	0.5	0.125	1	1	16
N. gonorrhoeae	CDC48	8	16	32	0.031	0.25	0.125	1	1	8
N. gonorrhoeae	CDC49	8	16	32	0.031	0.5	0.25	2	1	16
N. gonorrhoeae	CDC50	8	16	32	0.063	0.25	0.25	2	1	16
N. gonorrhoeae	CDC51	8	16	32	0.008	0.125	0.125	2	1	16
N. gonorrhoeae	CDC52	8	16	32	0.008	0.125	0.25	2	1	16
N. gonorrhoeae	CDC53	8	16	64	0.004	0.125	0.25	2	1	16
N. gonorrhoeae	CDC54	8	16	64	0.004	0.016	0.25	2	1	16
N. gonorrhoeae	CDC55	8	16	32	0.031	0.125	0.125	1	1	16
N. gonorrhoeae	CDC56	8	16	32	0.016	0.125	0.25	1	1	16
N. gonorrhoeae	CDC57	8	16	32	0.004	0.125	0.125	1	0.5	<0.016
N. gonorrhoeae	CDC58	8	16	32	0.004	0.125	0.25	2	1	16
N. gonorrhoeae	CDC59	8	16	32	0.004	0.125	0.25	2	16	4
N. gonorrhoeae	CDC60	8	16	32	0.031	0.125	0.125	4	1	16
N. gonorrhoeae	CDC61	8	16	64	0.031	0.25	0.125	4	1	32
N. gonorrhoeae	CDC62	16	16	32	0.008	0.125	0.25	0.25	0.5	0.125
N. gonorrhoeae	CDC63	8	8	32	<0.002	0.016	0.125	<0.008	0.063	<0.016
N. gonorrhoeae	CDC64	8	16	32	0.008	0.125	0.125	0.5	1	<0.016
N. gonorrhoeae	CDC65	8	16	32	0.016	0.125	0.125	0.5	0.5	<0.016
N. gonorrhoeae	CDC66	8	16	32	0.008	4	0.125	0.25	0.5	<0.016
N. gonorrhoeae	CDC67	8	16	64	0.008	0.125	0.125	0.5	1	0.25
N. gonorrhoeae	CDC68	8	16	32	0.016	0.125	0.125	1	1	16
N. gonorrhoeae	CDC69	8	16	32	0.031	0.125	0.063	1	1	16
N. gonorrhoeae	CDC70	8	16	32	0.004	4	0.125	2	1	16
N. gonorrhoeae	CDC71	8	16	32	0.008	0.125	0.016	0.25	1	0.25
N. gonorrhoeae	CDC72	8	16	32	0.031	0.125	0.25	2	1	16
N. gonorrhoeae	CDC73	8	16	32	0.008	0.125	0.125	0.5	1	0.125
N. gonorrhoeae	CDC74	8	16	32	0.008	0.125	0.125	0.25	1	<0.016
N. gonorrhoeae	CDC75	8	16	32	0.016	0.5	0.125	0.5	1	<0.016
N. gonorrhoeae	CDC76	8	16	64	0.016	0.125	0.125	0.5	1	<0.016
N. gonorrhoeae	CDC77	8	8	32	0.008	4	0.125	0.125	1	0.25
N. gonorrhoeae	CDC78	0.5	16	32	0.016	0.031	0.063	0.5	1	2
N. gonorrhoeae	CDC79	8	16	32	0.016	0.125	0.125	1	1	16
N. gonorrhoeae	CDC80	8	16	32	0.008	8	0.125	2	1	0.25
N. gonorrhoeae	CDC81	8	16	32	0.031	0.125	0.25	2	1	16
N. gonorrhoeae	CDC82	8	8	32	0.031	2	0.125	1	0.5	32
N. gonorrhoeae	CDC83	8	16	32	0.008	0.125	0.125	0.25	0.5	0.125
N. gonorrhoeae	CDC84	8	16	32	0.008	0.125	0.125	0.25	0.5	<0.016
N. gonorrhoeae	CDC86	8	8	32	0.008	0.125	0.125	<0.008	0.25	0.25
N. gonorrhoeae	CDC87	8	16	64	0.063	0.125	0.25	2	1	16
N. gonorrhoeae	CDC88	8	16	32	0.031	2	0.125	2	0.5	16
N. gonorrhoeae	CDC89	8	8	32	0.008	2	0.125	1	1	16
N. gonorrhoeae	CDC90	8	16	64	0.031	0.125	0.125	2	1	16
N. gonorrhoeae	CDC91	8	16	32	0.125	0.125	4	2	1	16
N. gonorrhoeae	CDC92	8	16	64	0.031	0.125	0.125	2	1	16
N. gonorrhoeae	CDC93	8	16	64	0.016	0.125	0.125	1	1	16
N. gonorrhoeae	CDC94	8	16	32	0.008	2	0.125	0.25	0.5	<0.016
N. gonorrhoeae	CDC95	8	16	32	0.031	0.125	0.25	2	1	16
N. gonorrhoeae	CDC96	8	16	32	0.016	0.125	0.125	1	0.5	16
N. gonorrhoeae	CDC97	8	16	32	0.031	0.125	0.125	1	1	16
N. gonorrhoeae	CDC98	8	16	32	0.008	2	0.125	0.25	0.5	<0.016
N. gonorrhoeae	CDC99	8	16	32	0.008	4	0.125	0.25	0.5	<0.016
N. gonorrhoeae	CDC100	8	16	32	0.031	0.125	0.25	1	1	16

APR, apramycin; AZI, azithromycin (S ≤ 1); CIP, ciprofloxacin (S ≤ 0.06); CIX, cefixime (S ≤ 0.25); CTR, ceftriaxone (S ≤ 0.025); GEN, gentamicin (S ≤ 16); PEN, penicillin (S ≤ 0.06); PRP, propylamycin; TET, tetracycline (S ≤ 0.25).

Table S3 Statistical significance (*p* values) of differences observed between the geometric mean MICs using the Mann-Whitney test (black font) and the Student's t-test (blue font).

	Gentamicin MIC = 4.9 µg/mL	Etimicin MIC = 3.7 µg/mL	Sisomicin MIC = 4.2 µg/mL	Netilmicin MIC = 3.2 µg/mL
Gentamicin		2.1e-05	0.00082	1.4e-08
Etimicin	2.1e-05		0.16173	0.04233
Sisomicin	0.0011	0.2539		0.00075
Netilmicin	1.7e-09	0.0385	0.0013	

Table S4 Frequency of *N. gonorrhoeae* aminoglycoside resistance

		Neissera gonorrhoeae		
		ATCC 49226	ATCC 19424	ATCC 43069
Gentamicin	8 µg/mL	4.3×10^{-7}	1.1×10^{-7}	$< 3.7 \times 10^{-9}$
Gentamicin	16 µg/mL	$< 7.8 \times 10^{-9}$	$< 6.9 \times 10^{-9}$	$< 3.7 \times 10^{-9}$
Gentamicin	32 µg/mL	$< 7.8 \times 10^{-9}$	$< 6.9 \times 10^{-9}$	$< 3.7 \times 10^{-9}$
Gentamicin C1a	8 µg/mL	3.9×10^{-7}	1.2×10^{-7}	$< 3.7 \times 10^{-9}$
Gentamicin C1a	16 µg/mL	$< 7.8 \times 10^{-9}$	$< 6.9 \times 10^{-9}$	$< 3.7 \times 10^{-9}$
Gentamicin C1a	32 µg/mL	$< 7.8 \times 10^{-9}$	$< 6.9 \times 10^{-9}$	$< 3.7 \times 10^{-9}$
Etimicin	8 µg/mL	$< 7.8 \times 10^{-9}$	$< 6.9 \times 10^{-9}$	$< 3.7 \times 10^{-9}$
Etimicin	16 µg/mL	$< 7.8 \times 10^{-9}$	$< 6.9 \times 10^{-9}$	$< 3.7 \times 10^{-9}$
Etimicin	32 µg/mL	$< 7.8 \times 10^{-9}$	$< 6.9 \times 10^{-9}$	$< 3.7 \times 10^{-9}$

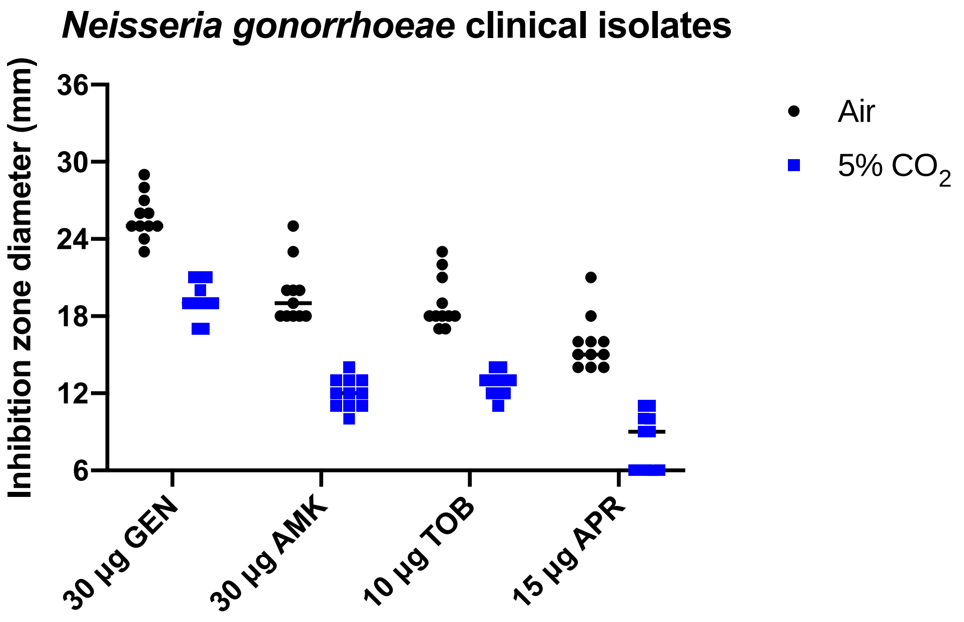

Fig. S1 Effect of atmosphere on the inhibition zone diameter.

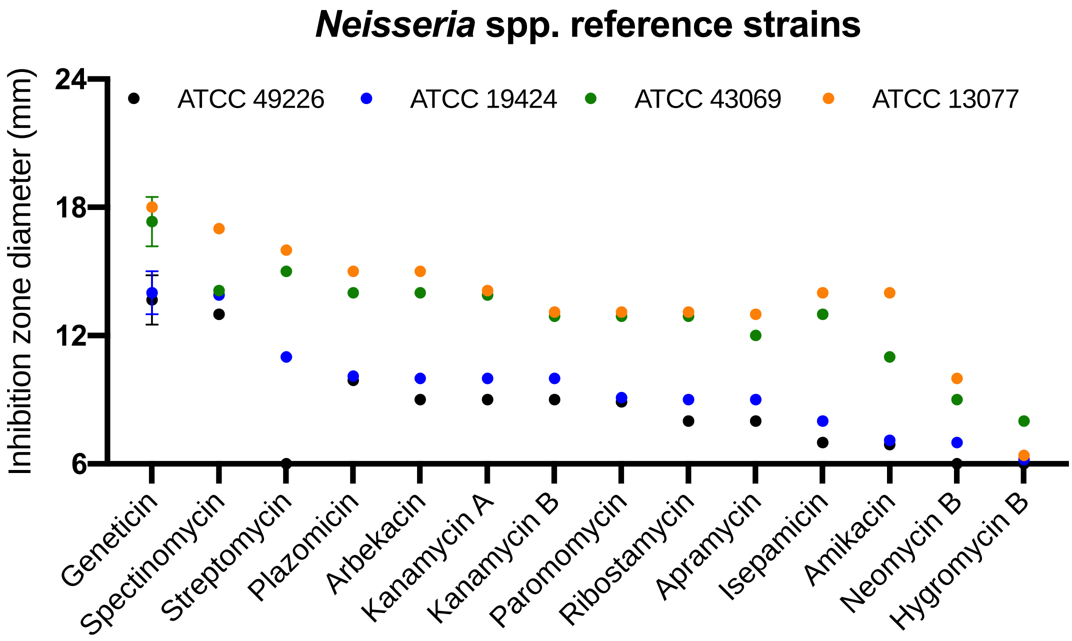

Fig. S2 In-vitro activities of other aminoglycoside antibiotics against *Neisseria* spp.

6-kanosamine-2-deoxystreptamines

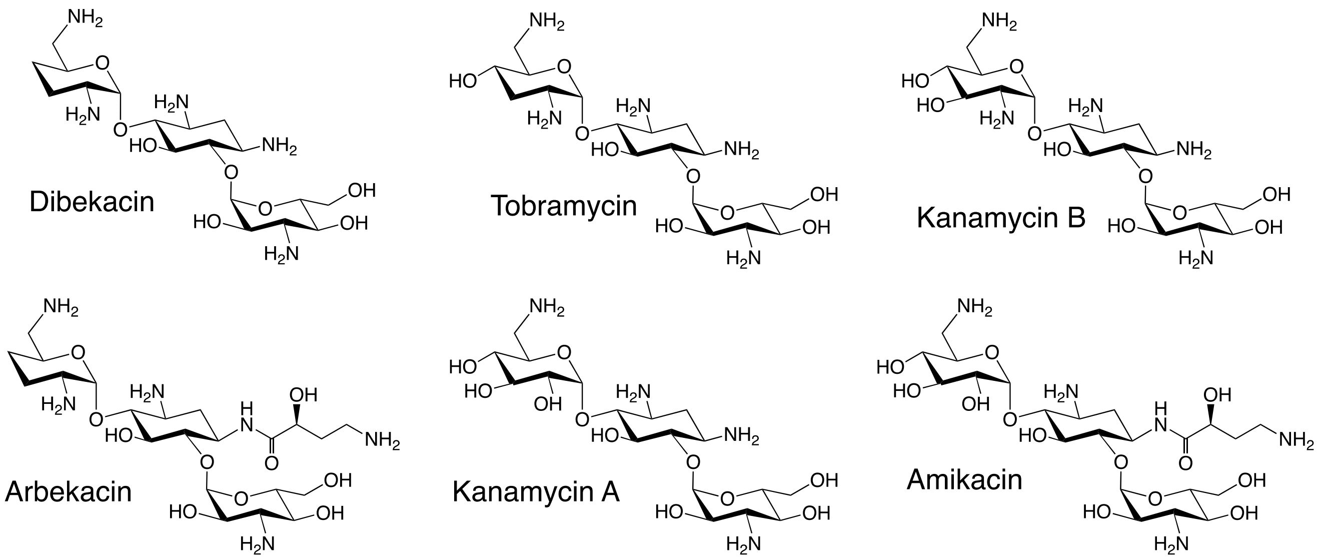

6-garosamine-2-deoxystreptamines

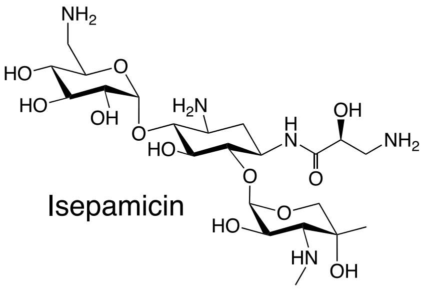

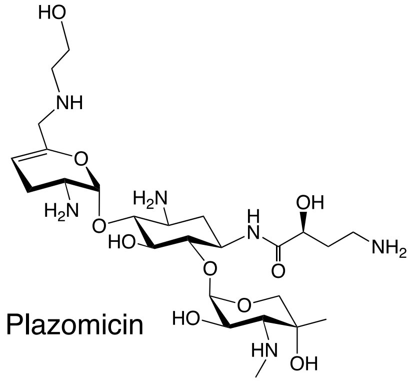

5-ribosyl-2-deoxystreptamines

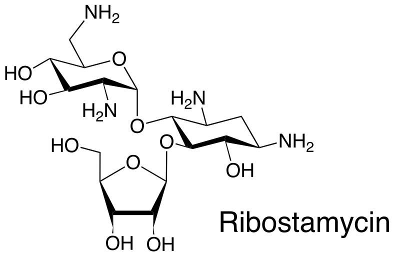

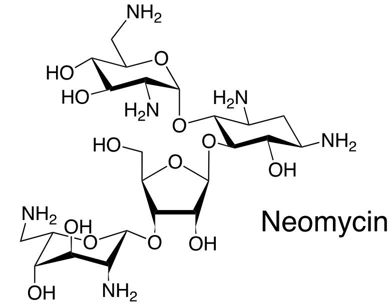

Fig. S3 Chemical structures of additional aminoglycosides discussed in the article.
